# Ocular-brain co-immunotherapy targeting amyloid-beta in Alzheimer’s disease: Challenges and promising therapeutic avenues

**DOI:** 10.4103/NRR.NRR-D-25-01399

**Published:** 2026-02-28

**Authors:** Xiaohong Xiang, Yong Tang, Linlin Song, Zhuohan Li, Betty Yuen Kwan Law

**Affiliations:** Dr. Neher’s Biophysics Laboratory for Innovative Drug Discovery, State Key Laboratory of Quality Research in Chinese Medicine, Faculty of Chinese Medicine, Macau University of Science and Technology, Macao Special Administrative Region, China; Department of Ophthalmology, Affiliated Hospital of Southwest Medical University, Luzhou, Sichuan Province, China; College of Integration of Traditional Chinese and Western Medicine & The Affiliated Traditional Chinese Medicine Hospital, Southwest Medical University, Luzhou, Sichuan Province, China

**Figure 1:**
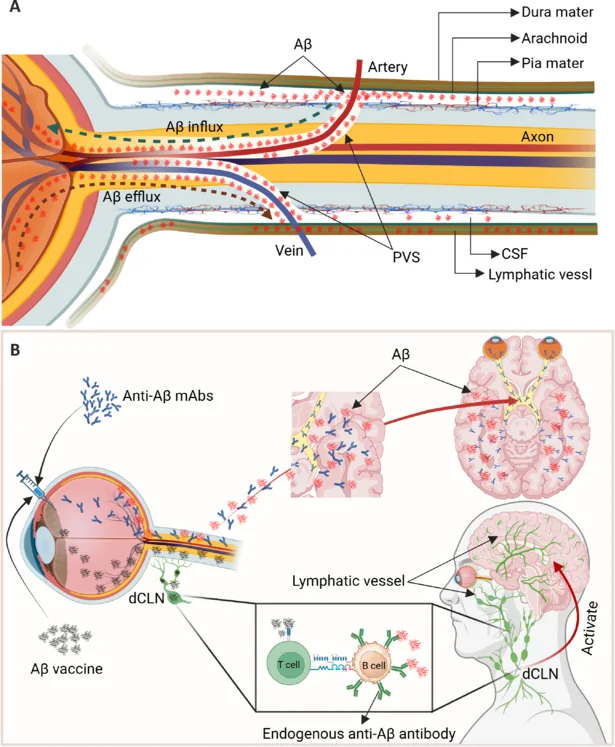
Influx and efflux of Aβ within the ocular system, alongside the co-immune targeting clearance of Aβ between the eye and the brain. (A) Intraocular influx and efflux of Aβ: Aβ is transported from the CSF in the subarachnoid space of the optic nerve sheath into the optic nerve axons, subsequently reaching the retina via the PVS of the artery. Aβ is then conveyed to the lymphatic system of the posterior ocular segment through the PVS of the vein. (B) Co-immune targeting clearance of Aβ in the eye and brain: Anti-Aβ mAbs are delivered to the retina via intravitreal injection, from where they travel to the brain through the optic nerve axons, facilitating the simultaneous clearance of Aβ from both the retina and the brain. Following intravitreal injection, the Aβ vaccine traverses the lymphatic vessels of the posterior ocular segment to reach the deep cervical lymph nodes. This process activates B cells to produce antibodies through T cell co-stimulation, thereby concurrently stimulating the ocular and cerebral immune systems to eliminate Aβ from the retina and brain. Created in BioRender. Qu, L. (2026) https://app.biorender.com/illustrations/689e9acea908c760b82521d1. Aβ: Amyloid-beta; anti-Aβ mAbs: anti-Aβ monoclonal antibodies; CSF: cerebrospinal fluid; dCLN: deep cervical lymph node; PVS: perivascular space.

**Alzheimer’s disease is an ocular-brain comorbidity:** Alzheimer’s disease (AD) is a leading neurodegenerative disorder affecting the central nervous system (CNS), clinically characterized by progressive cognitive decline, neuropsychiatric disturbances, and memory deficits. Around 60%–70% of dementia cases are due to AD, making it the most prevalent form. Current estimates suggest that approximately 50 million individuals globally are affected by AD, and the number of dementia patients is expected to rise to 139 million in 2050, with the cost of care projected to increase to nearly $1 trillion (Guo et al., 2024). The pathological hallmarks of AD predominantly consist of senile plaques, resulting from the accumulation of amyloid-beta (Aβ), and neurofibrillary tangles, arising from the hyperphosphorylation of tau protein. These features are accompanied by neuroinflammation, synaptic damage, and neuronal loss. AD is an ocular-brain comorbidity. During embryogenesis, both the retina and the brain develop from the neuroectoderm, resulting in shared anatomical, physiological, and embryological characteristics, including similarities in cell types, vasculature, and immune responses. The retina maintains structural and functional connections with the brain via the optic nerve, which links to the thalamus, optic radiation, and visual cortex. The deposition of Aβ has been observed in the postmortem eyes of AD patients and in the eyes of animal models of AD (Gaire et al., 2024). Aβ predominantly accumulates along blood vessels in the inner retinal layers, optic nerve axon bundles, meningeal lymphatic vessels of the optic nerve, and periorbital lymphatic vessels (Cao et al., 2024). Retinal Aβ is likely to originate from the brain rather than local retinal metabolism (Cao et al., 2024). Notably, Aβ can migrate from the brain to the eye within 60 minutes. The proposed mechanisms for the transport of Aβ from the brain to the eye encompass several pathways (**Figure 1A**): (1) Aβ transportation along the subarachnoid space and the optic nerve sheath, with entry into the optic nerve axons via perivascular spaces; (2) axonal transport within the optic nerve directed toward the proximal optic nerve; and (3) diffusion through the perivascular spaces of the central retinal artery into the retina (Cao et al., 2024). The clearance of Aβ in the eye involves multiple processes (**Figure 1A**): (1) Aβ uptake by retinal ganglion cells, followed by axonal transport through the lamina cribrosa and subsequent clearance via the meningeal lymphatic vessels of the optic nerve sheath and periorbital lymphatics; (2) glymphatic clearance facilitated by aquaporin-4 along the perivascular spaces; (3) phagocytosis and degradation by glial cells, including microglia and astrocytes; and (4) efflux across the blood–retina barrier into the systemic circulation for peripheral metabolism. The efficiency of Aβ clearance is modulated by various factors, including the cranio-ocular pressure gradient, expression levels of aquaporin-4, exposure to light, and the administration of cycloplegic agents (Wang et al., 2020).

Recently, the Lancet Commission has identified visual dysfunction as a modifiable risk factor for AD, highlighting its influence on cognitive function through mechanisms such as visual deprivation, social isolation, and vascular pathways (Livingston et al., 2024). Consequently, the retina is regarded as a window for monitoring the progression of AD. Due to their cost-effectiveness and high spatial resolution, various ophthalmic imaging technologies have been employed in the clinical assessment and monitoring of AD. Modified confocal scanning laser ophthalmoscopy with wide-field imaging, in conjunction with orally administered high-bioavailability curcumin, facilitates the noninvasive detection and quantification of Aβ in living AD patients. Optical coherence tomography provides rapid assessment of AD-specific retinal pathologies, including atrophy of the ganglion cell and inner plexiform layers, thinning of the retinal nerve fiber layer, and reductions in total macular and full retinal thickness. Optical coherence tomography angiography is utilized to assess retinal capillary density, including both the superficial and deep plexuses, as well as vascular tortuosity and macular flow indices. Multimodal ophthalmic imaging serves not only as a tool for monitoring ocular pathology but also provides valuable insights into cerebral Aβ burden, cerebrovascular status, and neurodegeneration. In comparison to positron emission tomography, cerebrospinal fluid, and blood-based biomarker detection methods for AD, ophthalmic imaging presents noninvasive, rapid, straightforward, and cost-effective advantages, rendering it particularly suitable for longitudinal monitoring of AD.

**Current status and challenges of immunotherapy targeting amyloid-beta in Alzheimer’s disease:** At present, there are no therapeutic agents available that can cure AD. The medications that have received clinical approval, such as cholinesterase inhibitors (rivastigmine, donepezil, and galantamine) and the non-competitive N-methyl-D-aspartate receptor antagonist (memantine), offer limited symptomatic relief but do not alter the trajectory of cognitive decline or halt disease progression. Immunotherapy targeting Aβ has become a prominent area of research in the treatment of AD, as it not only facilitates the clearance of Aβ aggregates but also shows promise in decelerating cognitive deterioration and disease progression. Active immunization strategies entail the administration of Aβ peptide vaccines to stimulate the endogenous production of anti-Aβ antibodies. The benefits of active immunization include sustained antibody production, cost-effectiveness, and a reduced frequency of injections. Several peptide vaccines, including CAD106, AN1792, ACI-24, and ABvac40, have shown varying degrees of efficacy in promoting Aβ clearance and preserving cognitive function (Sha et al., 2026). The effectiveness of this strategy depends on the patient’s cellular and humoral immune responses. Elderly patients with AD often exhibit immunosenescence, which can lead to suboptimal immune responses and reduced therapeutic efficacy. However, excessive production of Aβ antibodies may trigger harmful autoimmune reactions. Nonetheless, certain clinical trials have been discontinued due to adverse immune reactions, including pain at the injection site, aseptic meningoencephalitis, and changes in brain volume and mass. A significant challenge in active immunization is the optimization of vaccine formulations to ensure patient safety while maintaining sufficient immunogenicity and tolerability.

In contrast, passive immunotherapy involves the direct administration of exogenously synthesized anti-Aβ antibodies, which bind to cerebral Aβ deposits and facilitate their clearance by the immune system. This method eliminates Aβ without necessitating T-cell involvement, thereby circumventing T-cell-dependent inflammatory responses and minimizing adverse immune reactions. Monoclonal antibodies (mAbs) present distinct advantages in therapeutic development due to their high homogeneity, target specificity, and the immediate achievement of therapeutic antibody titers upon administration. Notably, three anti-Aβ mAbs have received approval from the U.S. Food and Drug Administration. Aducanumab, donanemab, and lecanemab are therapeutic agents that each target distinct epitopes or exhibit varying binding affinities to different forms of Aβ. While these therapies have had mixed results in removing Aβ, a new chapter in anti-amyloid treatment has commenced with the regulatory approvals of aducanumab in 2021, lecanemab in 2023, and donanemab in 2024. Aducanumab, the first human monoclonal antibody approved by the U.S. Food and Drug Administration, exhibits selective targeting of Aβ aggregates, including insoluble fibrils and soluble oligomers, through high-affinity binding to the N-terminus of Aβ. Clinical trials have demonstrated its efficacy in reducing cerebral Aβ burden and tau phosphorylation, while also decelerating cognitive decline in patients with early-stage AD (Budd et al., 2022). Lecanemab (BAN2401) is a humanized IgG1 monoclonal antibody that can bind to various forms of Aβ, such as oligomers and insoluble fibrils, with a specific focus on soluble Aβ aggregates. An 18-month treatment regimen resulted in a significant reduction in brain Aβ deposition and improved cognitive outcomes (van Dyck et al., 2023). Donanemab, another humanized IgG1 monoclonal antibody, recognizes the pyroglutamate-modified N-terminal epitope of Aβ, facilitating the selective clearance of established plaques. Clinical data indicate a 35.1% reduction in the rate of cognitive decline within 18 months, accompanied by 76.4% of patients treated with donanemab meeting the criteria for Aβ clearance within 76 weeks (Sims et al., 2023). The primary safety concern associated with these treatments is amyloid-related imaging abnormalities (ARIA), which occur at relatively high frequencies and present as headache, dizziness, falls, cerebral edema, microhemorrhages, or immune-mediated encephalitis (Salloway et al., 2025). Current therapeutic protocols necessitate administration every 2 or 4 weeks over a period of 12–18 months, incurring annual costs between $26,500 and $32,000, with limited insurance coverage. The high incidence of amyloid-related imaging abnormalities and the considerable financial burden pose significant obstacles to broad clinical adoption. Immediate priorities include optimization of antibodies to reduce adverse effects and formulation of cost-reduction strategies to enhance treatment accessibility for patients with AD.

**Ocular-brain co-immunotherapy — emerging therapeutic potential for Alzheimer’s disease:** The blood-brain barrier poses a significant impediment to drug penetration into the brain, presenting a substantial challenge in the development of therapeutics for AD. This challenge arises from various formidable obstacles related to effective drug delivery and the maintenance of therapeutic concentrations within the brain over extended periods. Anti-Aβ mAbs, which have molecular weights ranging from 144 to 150 kDa, illustrate this difficulty. The brain was thought to be an immune-privileged organ owing to the absence of a classical lymphatic drainage system in the parenchyma and the existence of the blood-brain barrier. Recent research into the shared immunity between the ocular and cerebral systems offers innovative solutions to this problem. Traditionally regarded as immune-privileged sites devoid of lymphatic drainage, both the eye and brain have recently been discovered to contain meningeal lymphatic vessels that play roles in fluid homeostasis, waste clearance, and immune surveillance. The glymphatic system contributes to the pathogenesis of various neurological disorders through its roles in clearing neurotoxic proteins, facilitating autoimmune cell infiltration, and transmitting pro-inflammatory signals, making it a critical therapeutic and preventive target for the CNS (Xu et al., 2023). Yin et al. (2024) demonstrated that intravitreal vaccination with herpes simplex virus induces protective immunity in the brain and revealed a shared lymphatic circuit capable of mounting a unified immune response between the eye and the brain. These results highlight an understudied immunological feature of the eye and open the potential for new therapeutic strategies in ocular and CNS diseases. We propose, for the first time, that anti-Aβ mAbs administered ocularly can induce co-immune targeted clearance of Aβ in the eye and brain for the treatment of AD (**Figure 1B**). Ocular delivery offers distinct advantages for AD therapy, providing dual therapeutic effects by addressing both ocular pathology and systemic AD symptoms. Enhanced CNS delivery can be achieved through potential mechanisms such as direct brain access via the optic nerve axonal transport system, activation of shared immune networks that promote cerebral Aβ clearance, and modulation of neuroinflammation through ocular cytokine networks. Pharmacokinetic benefits include bypassing the blood–brain barrier and hepatic first-pass metabolism, achieving higher concentrations in target tissues with reduced systemic exposure, and minimizing off-target effects while enhancing therapeutic precision. Clinically feasible administration methods encompass topical eye drops, which are non-invasive and exhibit excellent patient compliance. Intravitreal injections, which require doses 100 to 1000 times lower than those used in systemic administration, can greatly reduce drug costs. Conversely, the combination of intravitreal drug delivery and sustained-release pharmaceutical technology can further reduce medical costs. Fluocinolone acetonide intravitreal implants and aflibercept are two successfully developed intraocular sustained-release formulations that can continuously release drugs in the eye for 36 months and 6 months, respectively. Intraocular sustained-release pharmaceutical technology is also applicable to anti-Aβ mAbs, which will significantly reduce the frequency of administration and drug dosage. In addition to the development of intraocular sustained-release anti-Aβ mAb drugs, multi-target combination therapy for AD is also a potential research direction, such as combined Aβ and tau immunotherapy.

Translating this strategy into clinical practice faces several challenges. While intravitreal injection is a common and quick ophthalmology procedure, it carries risks such as hemorrhage, infection, increased intraocular pressure, and retinal detachment. Patients with mid-to-late-stage AD often struggle with cognitive decline and cooperation, complicating procedures under local anesthesia. In such cases, brief inhalation anesthesia is a viable alternative due to its minimal side effects. A major challenge is the complex physiological barriers and the unclear quantitative relationship between the eye and the brain. Developing an eye-brain pharmacokinetic model is essential to determine an effective intraocular dosage for the brain, requiring extensive clinical validation. Additionally, selecting the best drug delivery materials to extend dosing intervals remains a significant challenge. A key challenge in treating elderly AD patients is their age-related immune decline, which may reduce the effectiveness of active immunization. Passive immunization is less impacted, but using an Aβ vaccine via intravitreal administration might enhance immune response due to the brain-eye lymphatic connection. The main issue is managing ocular toxicity from anti-Aβ monoclonal antibodies, particularly ARIA-like events. These occur when antibodies rapidly clear amyloid deposits, compromising the vascular barrier and causing edema, microhemorrhages, and exudation. Due to the retina’s complex vascular network, addressing intraocular ARIA is crucial. Solutions involve finding a drug concentration that balances effectiveness and safety, and using sustained-release delivery systems to minimize damage from peak exposure. Additionally, ophthalmic multi-modality imaging offers a non-invasive way to monitor retinal neurovascular changes in real-time, allowing for timely treatment adjustments.

This perspective introduces, for the first time, the concept of ocular-brain co-immunotherapy for AD, utilizing unique neuro-ophthalmic connections to overcome traditional delivery barriers. Although questions remain regarding optimal dosing regimens and drug pharmacokinetics, this approach holds significant promise for the development of safer, more effective, and cost-efficient therapies for AD.
